# Low-cost VO_2_(M1) thin films synthesized by ultrasonic nebulized spray pyrolysis of an aqueous combustion mixture for IR photodetection

**DOI:** 10.1039/c9ra00189a

**Published:** 2019-03-29

**Authors:** Inyalot Jude Tadeo, Emma P. Mukhokosi, Saluru B. Krupanidhi, Arun M. Umarji

**Affiliations:** Materials Research Centre, Indian Institute of Science Bengaluru 560012 India umarji@iisc.ac.in +91-80-23607316 +91-80-22932944

## Abstract

We report detailed structural, electrical transport and IR photoresponse properties of large area VO_2_(M1) thin films deposited by a simple cost-effective two-step technique. Phase purity was confirmed by XRD and Raman spectroscopy studies. The high quality of the films was further established by a phase change from low temperature monoclinic phase to high temperature tetragonal rutile phase at 68 °C from temperature dependent Raman studies. An optical band gap of 0.75 eV was estimated from UV-visible spectroscopy. FTIR studies showed 60% reflectance change at *λ* = 7.7 μm from low reflectivity at low temperature to high reflectivity at high temperature in a transition temperature of 68 °C. Electrical characterization showed a first order transition of the films with a resistance change of four orders of magnitude and TCR of −3.3% K^−1^ at 30 °C. Hall-effect measurements revealed the n-type nature of VO_2_ thin films with room temperature Hall mobility, *μ*_e_ of 0.097 cm^2^ V^−1^ s^−1^, conductivity, *σ* of 0.102 Ω^−1^ cm^−1^ and carrier concentration, *n*_e_ = 5.36 × 10^17^ cm^−3^. In addition, we fabricated a high photoresponsive IR photodetector based on VO_2_(M1) thin films with excellent stability and reproducibility in ambient conditions using a low-cost method. The VO_2_(M1) photodetector exhibited high sensitivity, responsivity, quantum efficiency, detectivity and photoconductive gain of 5.18%, 1.54 mA W^−1^, 0.18%, 3.53 × 10^10^ jones and 9.99 × 10^3^ respectively upon illumination with a 1064 nm laser at a power density of 200 mW cm^−2^ and 10 V bias voltage at room temperature.

## Abstract 2

We report detailed structural, electrical transport and IR photoresponse properties of large area VO_2_(M1) thin films deposited by a simple cost-effective two-step technique.

## Graphical abstract

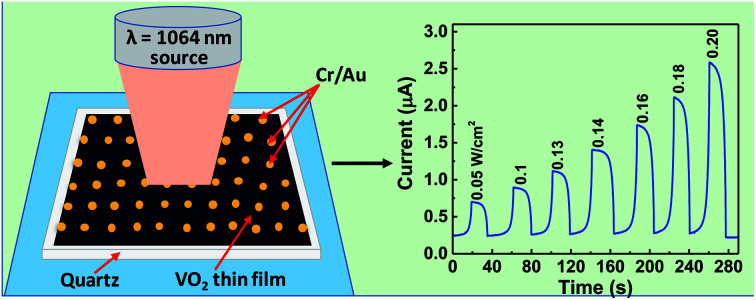

## 1. Introduction

Vanadium dioxide (VO_2_) is a phase change material that has been actively investigated due to its ability to undergo a reversible first order semiconductor-to-metal transition (SMT), also called a metal-to-insulator transition (MIT) at a temperature of about 68 °C accompanied with an abrupt resistance change of 4 to 5 orders of magnitude^1–3^ as well as changes in electrical, magnetic, optical, and transport properties.^4,5^ The change in physical properties is accompanied by the change in the crystal structure from the semiconducting monoclinic M1 phase (*P*2_1_/*c*) to a metallic tetragonal rutile (R) phase (*P*4_2_/*mnm*) characterized by a small lattice distortion along the *c*-axis.^1,3^ These exciting properties make VO_2_ appealing for a broad range of applications in electronics and opto-electronic switching devices such as field effect transistors (FETs),^4,6^ smart windows,^7,8^ micro-bolometers,^9^ thermal rectifiers^10^ and actuators.^11^ Transport properties such as carrier density and Hall mobility are important parameters in the Mott theory of MITs yet such data are scarce for VO_2_. As mentioned in the literature,^4^ Hall-effect measurements in VO_2_ are a challenging task due to low Hall mobility and unusually large amounts of noise ascribed to the strain present in the sample arising from the discontinuous lattice transformation at the structural phase transition.

Several methods, both physical and chemical, have been used to synthesize VO_2_ thin films. Physical methods among others include sputtering,^12^ pulsed laser deposition (PLD),^13^ molecular beam epitaxy (MBE),^14^ and electron beam evaporation.^15^ However, physical methods are expensive and produce thin films over a small area which does not favor industrial application. As such chemical methods of synthesis have been adopted to synthesize VO_2_ thin films over large area due to their low-cost and can easily be scaled up for industrial production. Some of these chemical methods include chemical vapor deposition (CVD),^16^ atmospheric chemical vapor deposition (APCVD),^17^ spray pyrolysis,^18–20^ sol–gel,^21^ and spin-coating.^22,23^ Ming Li *et al.*^24–27^ reported large scale synthesis of VO_2_(M) nanoparticles by combining hydrothermal synthesis with a subsequent mild thermal treatment. However, ultrasonic nebulized spray pyrolysis of aqueous combustion mixture (UNSPACM) gives a distinct edge over these other techniques because of its simplicity and robustness for depositing high quality thin films on large area without having to achieve stringent vacuum requirements.

Recently, Bharati *et al.*^28^ synthesized VO_2_ thin films on an expensive lanthanum aluminate (LAO) substrate by ultrasonic nebulized spray pyrolysis of aqueous combustion mixture (UNSPACM). For opto-electronic applications of VO_2_ thin films, material parameters such as structural, optical and electrical properties like carrier mobility and concentration are paramount. Here, we use a similar approach (UNSPACM) to synthesize high quality large area single phase VO_2_(M1) thin films on cheap and transparent quartz substrate for IR photodetection, an important application for both civilian and military such as high resolution imaging, light wave communication and optoelectronic circuits.^29^ Conventional commercial photodetectors such as InGaAS and Si usually require expensive substrates like sapphire and involve complex designs and cumbersome fabrication procedures. In this work, we present detailed structural, optical, electrical transport and IR photodetection properties of single phase VO_2_(M1) thin films. We demonstrate the potential of VO_2_(M1) thin films as a low-cost IR photodetector without going for complicated fabrication procedures. The fabricated IR photodetector exhibited good photoresponse with sensitivity, responsivity and detectivity of 5.18%, 1.54 mA W^−1^ and 3.53 × 10^10^ jones respectively upon illumination with a 1064 nm laser at a power density of 200 mW cm^−2^ and 10 V bias voltage at room temperature. We believe this article is of interest to the intended audience who will fabricate IR photodetectors based on this inexpensive technique.

## 2. Experimental

### 2.1. Synthesis

VO_2_ thin films in this work were obtained by a two-step process; (1) depositing V_2_O_5_ thin films on a pre-cleaned square (1 × 1 cm^2^) quartz substrate at 400 °C by ultrasonic nebulized spray pyrolysis of aqueous combustion mixture (UNSPACM) and then (2) reducing V_2_O_5_ thin films to obtain pure VO_2_ thin films using a similar approach reported elsewhere.^28^ UNSPACM combines solution combustion and spray pyrolysis techniques. The aqueous combustion mixture (ACM) consisting of an oxidizer (vanadyl nitrate) and a fuel (urea) in stoichiometric proportions was prepared by taking 1.2863 g of ammonium metavanadate and 1.6511 g of urea both from SD Fine-Chem Limited in 10 ml of distilled water. Concentrated nitric acid was then added drop-wise till a colorless solution was formed. Calculations were based on propellant reactions in which the oxidizer to fuel ratio was unity^30^ and the reaction equation given as below.

1 2VO(NO_3_)_3_ + 5CH_4_N_2_O → V_2_O_5_ + 8N_2_ + 10H_2_O + 5CO_2_

The ACM was taken into a specially designed glass set-up and nebulized using an ultrasonic medical nebulizer (Mystique Air Sep USA) of 2.5 MHz frequency; a schematic diagram of the nebulized spray pyrolysis set-up is given elsewhere.^31^ The ultrasonically nebulized mist containing the redox mixture was carried to the hot substrate by N_2_ gas flowing at 1000 sccm. The micrometer sized droplets instantaneously pyrolyzed on reaching quartz substrate maintained at 400 °C. The films were deposited for 10 minutes. The oxide phase forms only after the droplets reached the hot substrate due to highly exothermic self-propagating reaction of the redox mixture. The optimized deposition parameters were arrived at after mapping out different variables. The chemical reaction yielded V_2_O_5_ which was then reduced to VO_2_ by a reduction technique reported elsewhere.^28^ The conditions for reduction such as temperature and partial pressure of oxygen were arrived at with the help of the V–O phase diagram and the oxygen partial pressure *versus* temperature plot^1,32^ such that single phase VO_2_ would result.

### 2.2. Characterization

Structural characterization of the films was carried out using X-ray diffraction (XRD) X'Pert-PRO PANalytical instrument with Cu-Kα radiation (1.5418 Å) at a scan rate of 2° per minute. Room temperature (RT) and temperature variable (RT-to-70 °C) Raman spectra of the films were recorded from 50–1100 cm^−1^ on a Horiba JobinYvon HR-Raman-123 microPL spectrometer with a wavelength of 532 nm. The surface morphology of the thin films was analyzed using non-contact mode atomic force microscope (A.P.E. Research A100-AFM) and using Inspect F50 field emission scanning electron microscope (FESEM). The film thickness was measured using Veeco Dektak 6M surface profilometer. UV-visible measurements were carried out on the thin films using UV-vis-NIR spectrophotometer (Perkin Elmer-Lambda 750 instrument). The VO_2_ thin films were further characterized using a Fourier transform infrared (FTIR) spectrometer (Agilent Cary 660) coupled with an IR microscope (Cary 600). The reflectivity from the sample was characterized as a function of substrate temperature. A stage with temperature control was used to heat the sample at 5 °C increments and the reflection spectra collected at respective temperatures. The reflectance was normalized to the reflectivity of a plain gold mirror. To investigate the chemical electronic states of the prepared samples, X-ray photoelectron spectroscopy (XPS) measurements were carried out on an Axis Ultra DLD (from Kratos) high resolution instrument with automatic charge neutralization equipped with Mg-Kα radiation (1253.5 eV). The data was fitted using XPS Peak41 software.^28^

### 2.3. Device fabrication

The IR photodetector device based on VO_2_(M1) was fabricated by depositing electrical contacts of Cr/Au (6 nm/80 nm) by thermal evaporation using a mask placed on top of VO_2_ thin films. The contacts were 1 mm wide and 1 mm apart. The (*I*–*V*) measurements on VO_2_ thin films were carried out on a DC probe station equipped with an ATT thermal controller coupled with a B1500A semiconductor device analyzer. The photodetection characteristics of the device were measured using a Keithley SMU2400 source meter and 1064 nm laser with varying intensity. Hall-effect measurements were conducted from room temperature to 80 °C using Ecopia HMS 5000 Hall-effect measurement system. Four equally spaced ohmic contacts were selected and measurements were taken in Van der Pauw geometry in the presence of 0.55 T magnetic field.

## 3. Results and discussion

### 3.1. X-ray diffraction

**Fig. 1:**
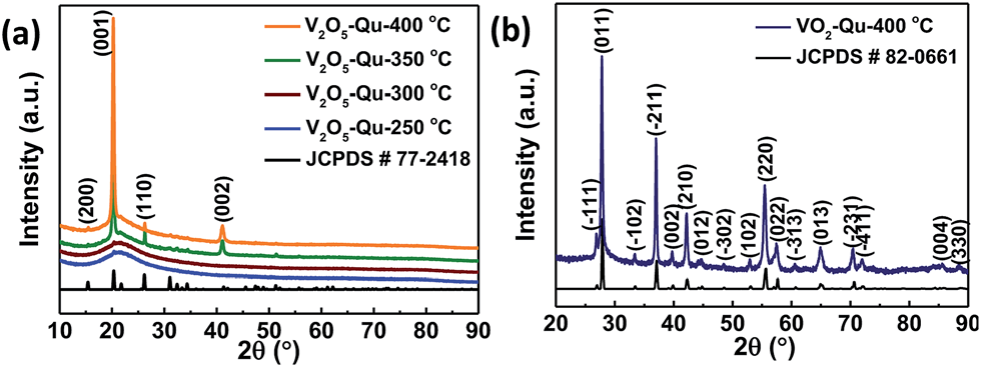
X-ray diffraction (XRD) patterns of (a) V_2_O_5_ thin films deposited on quartz substrate at 250 to 400 °C, (b) VO_2_ thin films deposited on quartz substrate at 400 °C by UNSPACM.

Fig. 1a shows the X-ray diffraction (XRD) pattern of a 420 ± 10 nm thick V_2_O_5_ thin films deposited on quartz substrate at different temperatures (250 °C to 400 °C) by UNSPACM. The optimized deposition temperature of 400 °C was arrived at by experimentally employing various substrate temperatures (250–400 °C). Films deposited at 250 °C and 300 °C were amorphous while films deposited at 350 °C and 400 °C were crystalline. Best crystallinity was exhibited by films deposited at 400 °C. The V_2_O_5_ thin films were later reduced to VO_2_ thin films which was confirmed from its XRD pattern shown in Fig. 1b. In both cases pure phases of V_2_O_5_ and VO_2_ were obtained with no detectable peaks due to any other vanadium oxide phase. The strong intensity of the (001) plane of V_2_O_5_ thin film coupled with the extinction of most (*hkl*) planes in comparison to the XRD pattern of powder V_2_O_5_ suggests a high preferential orientation of V_2_O_5_ thin films along the *c*-axis. V_2_O_5_ was indexed to orthorhombic crystal system of space group *Pmmn* using JCPDS # 77-2418 (ref. 33) while VO_2_ was indexed monoclinic (M1) phase of VO_2_ with space group *P*2_1_/*c* (JCPDS # 82-0661).^34,35^

### 3.2. Raman spectroscopy

**Fig. 2:**
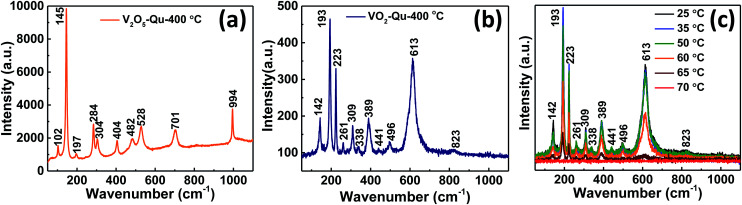
Room temperature Raman spectra of (a) V_2_O_5_ and (b) VO_2_ thin films, (c) temperature dependent Raman spectra of VO_2_ thin films deposited on quartz substrate at 400 °C by UNSPACM.

The phase purity of V_2_O_5_ and VO_2_ thin films was further confirmed from Raman spectra measurements, it being a very sensitive tool in detecting local structural variations.^36^Fig. 2a represents the room temperature Raman spectra of V_2_O_5_ thin films deposited on quartz substrate by UNSPACM. V_2_O_5_ structure can be described from the packing of V_2_O_5_ layers along the *c* axis of the unit cell. The unit cell contains two formula units.^32^ Each layer is built up from VO_5_ square pyramid units which share edges thus building double chains along the *b*-direction.^37^ The chains are connected by their corners and this results into octahedrally coordinated VO_6_ with three different principal V–O distances leading to three different oxygens;^32,38^ the very short (1.58 Å) terminal (V–O_1_) double bond along the *c* direction, the doubly (V–O_2_) coordinated and triply (V–O_3_) coordinated bridging oxygen in the basal plane (1.77–2.02 Å) and weak V–O (2.79 Å) bonds in between the layers.^32,38^ Under the *D*_2h_ factor group (*k* = 0), the crystal modes of V_2_O_5_ can be classified as follows:^39^*Γ*_opt_ = 7A_g_ + 7B_1g_ + 3B_2g_ + 4B_3g_ + 3A_u_ + 3B_1u_ + 6B_2u_ + 6B_3u_

All g modes are Raman active, while only B_u_ modes are IR active. 3A_u_ modes are inactive according to the selection rule for *D*_2h_ space group. Therefore 21 Raman and 15 IR active modes are expected for V_2_O_5_. The Raman spectra of V_2_O_5_ thin films featured 10 peaks at wavenumbers 102(A_g_), 145(B_3g_, B_2g_), 197(B_1g_), 284(B_2g_, B_3g_), 304(A_g_), 404(A_g_), 482(A_g_), 528(A_g_), 701(B_2g_, B_3g_) and 994(A_g_) cm^−1^, which correspond to the wavenumber values reported for oriented crystalline V_2_O_5_.^38^ The peak located at about 102 cm^−1^ is attributed to the external T_y_ modes.^39^

The predominant peak at 145 cm^−1^ is attributed to the skeleton bent vibration of the V–O–V bond and its presence is an evidence for the layered structure of V_2_O_5_ thin film.^39,40^ The peaks at 197 and 284 cm^−1^ are from the bending vibration modes of the O_3_–V–O_2_ bond.^38^ The peak at 404 cm^−1^ is assigned to the bending vibration mode of the V–O_2_–V bond.^38^ The two peaks located at 482 and 304 cm^−1^ are assigned to the bending vibration modes of bridging doubly coordinated oxygen (V–O–V) bond and triply coordinated oxygen (V–O_3_) bond respectively.^40^ The peak at 528 cm^−1^ is assigned to the triply coordinated oxygen (V–O_3_) stretching vibration mode which results from edged-shared oxygens in common to the three pyramids.^39,40^ Another peak located at 701 cm^−1^ is assigned to the doubly coordinated oxygen (V–O_2_–V) stretching vibration mode which results from corner-shared oxygens common to the pyramids.^38,40^ And the high-frequency Raman peak located at about 994 cm^−1^ is assigned to the terminal oxygen (V=O) stretching vibration mode which results from unshared oxygen.^38,40^Fig. 2b shows room temperature Raman spectra of the synthesized VO_2_ thin films. It clearly shows the distinct M1 phase of VO_2_ as the peaks identified match well with M1 phase reported.^6,41^ We identified 11 peaks at wavenumbers 142, 193, 223, 261, 309, 338, 389, 441, 496, 613 and 823 cm^−1^ and were consistent with literature reports for VO_2_.^6,15,40,41^ The peaks were assigned as B_1g_, A_g_, A_g_, A_g_, A_g_, A_g_, A_g_, E_g_, A_g_, A_g_ and B_2g_ respectively phonon modes of M1 monoclinic VO_2_.^6,29^ The Raman spectra of VO_2_ are in three sets of V–O modes; bands at low wavenumbers (<400 cm^−1^) are assigned to V–O–V bending modes, those at intermediate wavenumbers (400–800 cm^−1^) are assigned to the V–O–V stretching modes and bands at high wavenumbers (>800 cm^−1^) are assigned to V=O stretching modes of distorted octahedra and distorted square-pyramids.^36^ The phonon modes of VO_2_(M1) are composed of stretching and bending modes of V–O–V bond and zigzag chains of V–V.^6,36^ To further study the phase structure and phase transition, Raman measurements were performed at different temperatures as shown in Fig. 2c. The intensity of all the 11 peaks observed at room temperature which characterize the presence of M1 phase^6,15,40,41^ decreased with increase in temperature and completely vanished at high temperature (70 °C). The Raman spectra became broad band which indicates the formation of VO_2_ rutile (R) phase; transforming from the M1 phase.^42,43^ The sudden flattening of the Raman peaks at high temperature, above the transition temperature of VO_2_ (68 °C),^42^ is indicative of switching at that temperature and thereby reflecting the high quality of our thin films. The peaks reappeared when the temperature was reduced towards room temperature confirming the reversible switching of the films above and below their transition temperature. The results were repeating for multiple circles of heating and cooling. The change in the Raman spectra of VO_2_ thin films compared to that of V_2_O_5_ thin films further confirmed the reduction of V_2_O_5_ to VO_2_.

### 3.3. Surface morphology

**Fig. 3:**
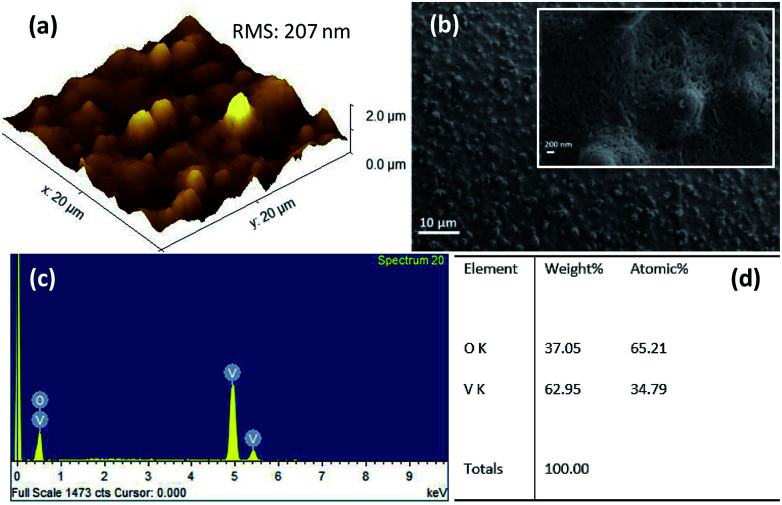
(a) 3D AFM image, (b) SEM image; inset is high magnification SEM image, (c) EDX spectrum, and (d) elemental composition of VO_2_ thin films deposited on quartz substrate at 400 °C by UNSPACM.

The surface morphology of the films was investigated using non-contact atomic force microscope (AFM) and field emission scanning electron microscope (FESEM). Fig. 3a shows 3D AFM image of VO_2_ thin films deposited on quartz substrate at 400 °C by UNSPACM. It clearly shows the influence of combustion during deposition process on the surface morphology of the deposited films. The films had a high root mean square roughness (rms) value of 207 nm as expected for films synthesized by UNSPACM technique.^28,31^ The SEM image of VO_2_ thin films shown in Fig. 3b reveals the porous nature of the films. The high magnification image of the films given by the inset of Fig. 3b reveals the continuous well grown and packed grains of 30–50 ± 0.05 nm suitable for this application. This unique morphology of the films results due to the impact of micro size particles which undergo rapid combustion reaction on the hot quartz substrate and the formation of large amounts of gaseous products (23 moles of gases are evolved per mole of V_2_O_5_ formed) from a violet combustion reaction like volcanic eruption.^31^

The energy dispersive X-ray spectroscopy (EDX) spectrum obtained from the SEM is given in Fig. 3c; it confirms the presence of vanadium (V) and oxygen (O) in the sample. The stoichiometric composition of VO_2_ was confirmed from the elemental composition given in Fig. 3d.

### 3.4. X-ray photoelectron spectroscopy

**Fig. 4:**
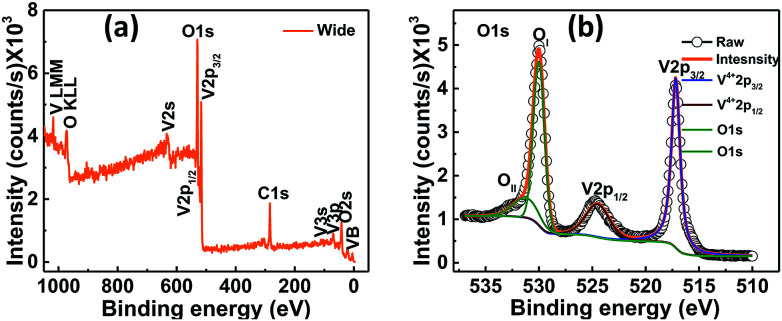
XPS spectra for VO_2_ thin films deposited on quartz substrate at 400 °C by UNSPACM (a) survey spectrum and (b) O 1s and V 2p region.

The electronic states of the synthesized VO_2_ thin films on quartz substrate were studied by X-ray photoelectron spectroscopy (XPS) and chemical states of the sample were investigated. In the survey spectrum, peaks corresponding to O 1s, V 2p_3/2_ and V 2p_1/2_ were identified as shown in Fig. 4a.

The binding energy (BE) of the adventitious C 1s peak was taken as 284.8 eV. The O 1s and V 2p peaks were calibrated based on the BE of the adventitious C 1s peak. The background subtraction was carried out using Shirley background function (Fig. 4b). The binding energy of V 2p_3/2_ peak was found to be 517.1 eV with FWHM of 0.97 eV while the BE of the V 2p_1/2_ peak was found to be 524.4 eV with FWHM of 2.6 eV as shown in Fig. 4b. Both peaks were assigned to V^4+^ oxidation state^15,44^ confirming the stoichiometry of VO_2_. No other oxidation states of vanadium were detected. High energy peaks relate to O 1s energy level. The peak at 530.0 eV with FWHM of 1.2 eV is assigned to O 1s of the native oxygen (that is from the O–V bond in the sample) whereas the peak at 531.2 eV with FWHM of 2.8 eV is attributed to the oxygen arising from the surface contamination by atmospheric CO_2_ or H_2_O.^15^

### 3.5. Optical

**Fig. 5:**
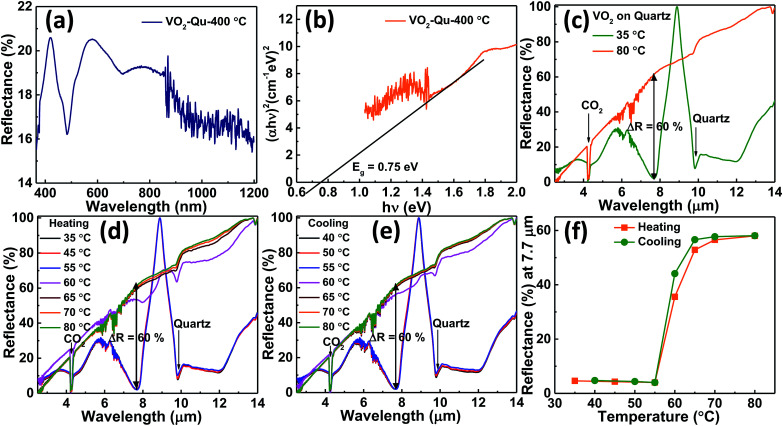
(a) DRS (b) Tauc plot for VO_2_ thin films deposited on quartz substrate at 400 °C by UNSPACM, (c) measured reflectance from VO_2_ thin films on quartz substrate at 35 °C and 80 °C (d) on heating, (e) on cooling and (f) at *λ* = 7.7 μm of wavelength during heating and cooling.

The optical properties of VO_2_ thin films were studied from the diffuse reflectance (DRS) measurements taken from 200–1200 nm of wavelength as shown in Fig. 5a. The DRS was converted to equivalent absorption spectra using Kubelka–Munk (KM) function.^45–48^ The KM function at any wavelength is given by *F*(*R*_∞_) = (1 − *R*_∞_)^2^/2 *R*_∞_ = *α*/*s* where *R*_∞_ is the reflectance of the film relative to reference material *i.e.* (*R*_sample_/*R*_reference_), *α* is the absorption coefficient and *s* is the scattering coefficient. The scattering coefficient is weakly dependent on energy, therefore *F*(*R*_∞_) is assumed to be proportional to the absorption.^48^ The optical band gap of VO_2_ thin films was then estimated to be 0.75 ± 0.01 eV from the Tauc plot^49–51^ by plotting (*αhν*)^2^ against *hν* for direct band gap and then extrapolating the linear portion of curve to the horizontal axis as shown in Fig. 5b.

Our results are in good agreement with the reported band gap values, 0.6–1.0 eV (ref. 12, 52–55) for semiconducting (monoclinic) VO_2_. The reflectance spectra for VO_2_ thin films on quartz substrate was recorded as a function of substrate temperature using FTIR spectrometer (Agilent, Carry 660 coupled with Carry 600 IR microscope). The spectral response was measured from 2.5 μm to 14 μm IR frequencies since the surface plasmon in VO_2_ metallic phase is only observed at *λ* > 2 μm.^56^Fig. 5c shows the reflectance of the low temperature (below 68 °C) semiconducting phase and high temperature (above 68 °C) metallic phase of VO_2_ thin films. The metallic phase was highly reflecting as compared to the semiconducting phase. This was consistent with literature reports on reflectance change^7,57^ for VO_2_ thin films across the transition. At the semiconducting state (temperatures below 68 °C), the V atoms of VO_2_ pair up and open energy gap^58^ (0.75 eV, as shown in Fig. 5b) permitting high IR transmission^7,59^ whereas in the metallic state (temperatures above 68 °C), the overlap between the Fermi level and the V_3d_ band eliminates the above mentioned band gap,^60,61^ causing the material to be highly reflecting in the NIR region.^7,62^ At *λ* = 7.7 μm of wavelength, reflectivity of VO_2_ thin film changed from about 2% in the semiconducting phase to about 62% in the metallic phase exhibiting the reflectance change, Δ*R* of about 60%. This was reversible and repeatedly seen in many heating and cooling cycles shown in Fig. 5d and e respectively. The sharp dip in reflectance observed at *λ* = 4.3 μm is due to the presence of atmospheric CO_2_ (ref. 56) which interferes with the measurements while the dip observed at *λ* = 9.8 μm is attributed to the substrate (quartz). The reflectance at *λ* = 7.7 μm of wavelength for various heating and cooling temperatures extracted from Fig. 5d and e respectively was plotted as shown in Fig. 5f. It shows the reflectance change of VO_2_ thin film across the phase transition.

### 3.6. Electrical

**Fig. 6:**
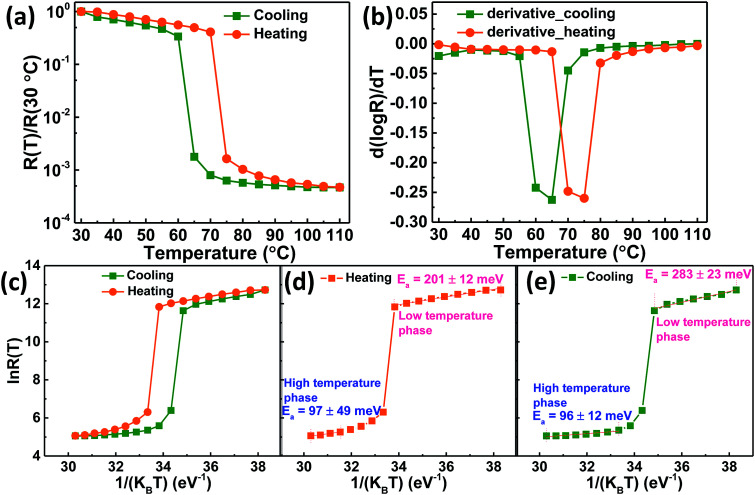
(a) Normalized resistance as a function of temperature, (b) derivative d(log *R*)/d*T* plot for heating and cooling, (c) activation energy analysis from ln *R*(*T*) *versus* 1/*K*_B_*T* plot for heating and cooling, (d) heating alone and (e) cooling alone, for VO_2_ thin films deposited on quartz substrate at 400 °C by UNSPACM.

The electrical properties of VO_2_ thin films deposited on quartz substrate was determined by in-plane geometry measurements at various temperatures using two probe DC probe station equipped with an ATT thermal controller coupled with a B1500A semiconductor device analyzer. Electrical contacts, Cr/Au (6 nm/80 nm) deposited on top of VO_2_ thin films (1 mm wide and 1 mm apart) by thermal evaporation were linear and ohmic in nature. The current–voltage (*I*–*V*) measurements for VO_2_ thin film were then carried out at various temperatures from which the resistance (*R*) was obtained from the slope of *V versus I* plot. Fig. 6a shows the temperature dependence of normalized resistance, *R*(*T*)/*R*(30 °C). It shows an abrupt first order transition of four orders of magnitude. The insulator-metal transition temperature (*T*_IMT_) for VO_2_ thin film was determined from the intersection of heating and cooling *R*–*T* derivative curves in Fig. 6b. It was found to be 68 °C which agrees with literature reports.^2,53^ The transition width (Δ*T*), which is the full width at half maximum of the derivative curve and the thermal hysteresis (Δ*T*_h_), defined as the difference between the critical temperature during heating and that during cooling^28^ for VO_2_ thin films were found to be 10 and 9 °C respectively, smaller than the literature^1,28,62^ values. The resistance ratio, expressed as Δ*A* = *R*(30 °C)/*R*(110 °C), gives the strength of IMT was found to be 2122 and 2140 for heating and cooling cycles respectively.

This huge Δ*A i.e.* more than three orders of magnitude, could be attributed to the complete formation of M1 phase of polycrystalline VO_2_ thin film without any other inter-grain phases. The temperature coefficient of resistance (TCR) of VO_2_ thin film was calculated and found to be −3.3% K^−1^ at 30 °C; the result matching well with literature reports.^63,64^Fig. 6c shows the ln *R*(*T*) *versus* 1/*K*_B_*T* plot for semiconducting and metallic phases. Activation energy, *E*_a_ was deduced from *R*(*T*) = *R*_o_ exp(*E*_a_/*K*_B_*T*), where activation energies are the slopes of ln *R*(*T*) *versus* 1/*K*_B_*T* plot. *E*_a_ was determined for both heating and cooling in Fig. 6d and e respectively. It was found to be 201 ± 12 meV in the semiconducting phase and 97 ± 49 meV in the metallic phase during heating, whereas during cooling it was found to be 96 ± 12 meV in the metallic phase and 283 ± 23 meV in the semiconducting phase. These results were comparable with the reported values.^54,65^

### 3.7. Hall-effect measurements

**Fig. 7:**
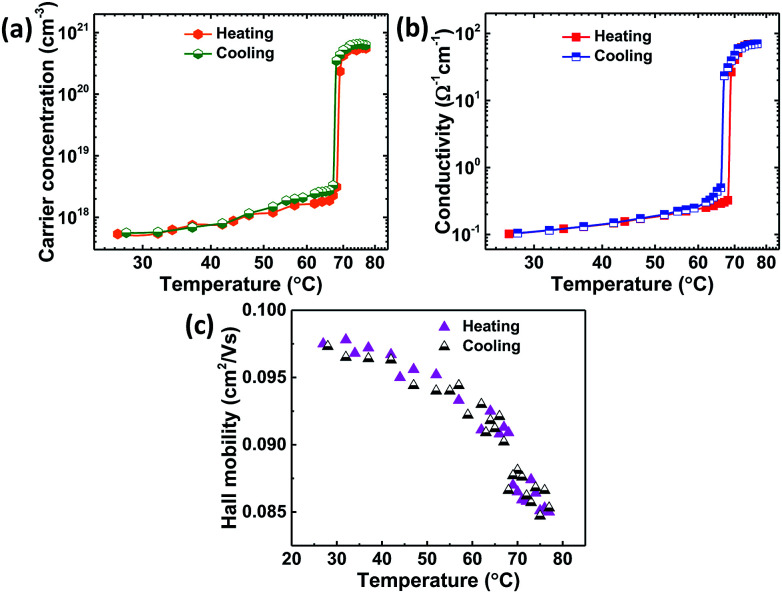
Transport properties as a function of temperature from Hall-effect measurements of VO_2_ thin films (a) carrier concentration, (b) conductivity and (c) Hall mobility.

To investigate transport properties of VO_2_ thin films deposited on quartz substrate, temperature-dependent (27–77 °C) Hall-effect measurements were carried out across the insulator to metal transition (IMT) of as-deposited VO_2_ thin films as shown in Fig. 7. The maximum temperature of 77 °C was limited by our instrument. Electrons were found to be the majority carriers both in the semiconducting and metallic phase revealing the n-type nature of VO_2_ films and was consistent with the literature reports.^4,51,54,66^ The films showed room temperature mobility, *μ*_e_ of 0.097 cm^2^ V^−1^ s^−1^, conductivity, *σ* of 0.102 Ω^−1^ cm^−1^ and carrier concentration, *n*_e_ = 5.36 × 10^17^ cm^−3^ which is in a good agreement with other literature reports.^4,16,54^ The temperature dependence of carrier concentration (Fig. 7a) shows an abrupt increase by three orders of magnitude from 5.36 × 10^17^ cm^−3^ at 27 °C to 5.56 × 10^20^ cm^−3^ at 77 °C which accounts for the decrease in resistivity manifested by the increase in conductivity (Fig. 7b) by three orders of magnitude. In metallic state, the conductivity is relatively temperature-independent due to nearly constant carrier concentration.

The temperature dependence of Hall mobility (Fig. 7c) shows that it slowly decreases with temperature (approximately as *T*^−3/2^) which can be attributed to scattering of band electrons from acoustic phonons and was consistent with other reports.^4,54,66,67^

### 3.8. Photodetection

**Fig. 8:**
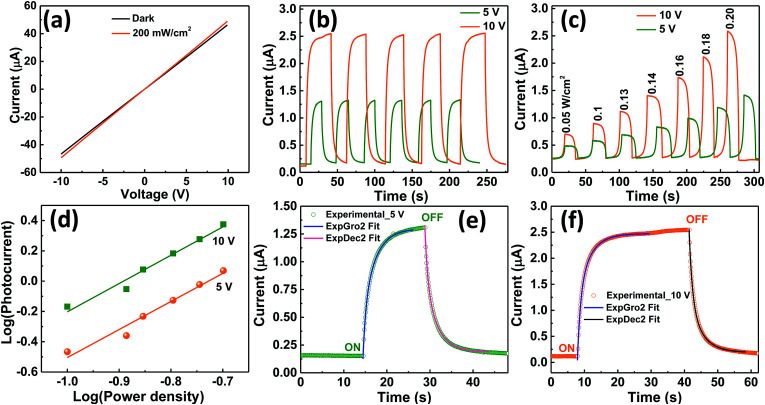
(a) *I*–*V* for the fabricated VO_2_ device under the dark and on illumination with IR (1064 nm) laser (b) ON and OFF IR response characteristic of the device at 5 and 10 V bias voltage for several cycles (c) IR photoresponse of the film with varying power densities under bias voltage of 5 and 10 V (d) photocurrent *versus* power density (*I*_photon_ ∝ *P*^m^) plot, (e) experimental and fitted growth and decay curves at 5 V bias voltage and (f) experimental and fitted growth and decay curves at 10 V bias voltage.

The optoelectronic properties of the fabricated device based on VO_2_(M1) thin films were investigated under illumination with IR (1064 nm) laser at different radiation intensities. Fig. 8a shows the room temperature *I*–*V* curves of the photodetector in the dark and under illumination with a 1064 nm laser with bias voltage ranging from −10 V to 10 V. The curves are linear confirming ohmic nature of the contacts. Fig. 8b shows the photocurrent change curves with respect to time at bias voltage of 5 and 10 V under constant power density of 200 mW cm^−2^. It was observed that photocurrent increases with increase in bias voltage with good repeatability and stability for several cycles. Photocurrent was given by *I*_ph_ = *I*_light_ − *I*_dark_^68,69^ where *I*_light_ is the current on illumination by the laser and *I*_dark_ is the dark current. A photocurrent of 1.17 μA was generated by the device upon illumination under 1064 nm laser with power density of 200 mW cm^−2^ at 5 V bias voltage and a much higher photocurrent of 2.43 μA was generated when the bias voltage was increased to 10 V.

**Fig. 9:**
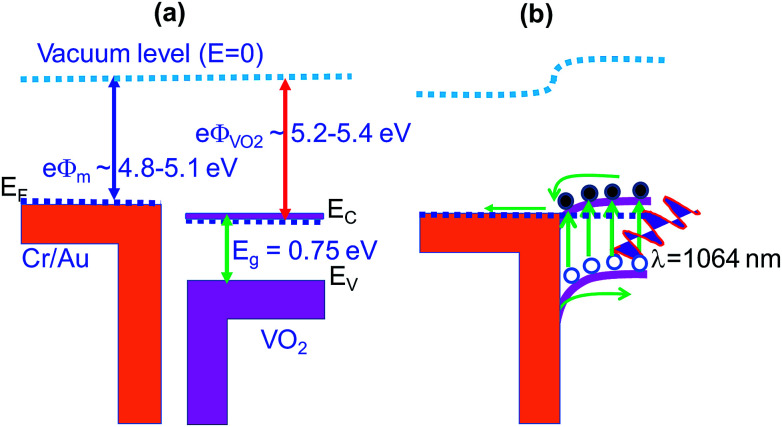
Energy band alignment of Cr/Au and VO_2_ (a) before contact and (b) at contact.

The photocurrent was also found to increase with increase in power density as shown in Fig. 8c. The dependence of photocurrent on power density is shown in Fig. 8d. It is fitted with the power law: *I*_ph_ ∝ *P*^*m*^ where *I*_ph_ is photocurrent, *P* is the power density and *m* is exponent which determines the response characteristic of a photodetector with incident power density.^70^ Its value was found to increase from 0.73 to 0.89 when the bias voltage was increased from 5 to 10 V. This indicates that trap states and interactions between the photo-generated carriers (electron–hole pairs) are involved in the recombination kinetics of the photo-carriers^45^ at lower bias and this value is close to unity at higher bias whereby the photo-generated current can be attributed to better separation of electron–hole pairs with less trap states and interaction between photo-generated charge carriers. The deviation of *m* value from unity, the ideal slope value is attributed to defects^69^ or charge impurity states^45^ in the sample and the recombination-effect which leads to loss of photocarriers. The working principle of the device can be explained using the energy band alignment of Au and VO_2_ shown in Fig. 9. The *I*–*V* characteristics of the fabricated metal–semiconductor interface is ohmic as expected since the work function of VO_2_ (5.2–5.4 eV) is greater than the work function of Au (4.8–5.1 eV).^71–73^ The schematic of isolated metal and VO_2_ before contact is depicted in Fig. 9a. When the metal and the semiconductor are brought into contact as shown Fig. 9b, electrons flow from metal to VO_2_ until the Fermi-levels are aligned. The barrier between the metal and VO_2_ is so small that it can easily be overcome by a small voltage. When the device is illuminated with IR (*λ* = 1064 nm ∼ 1.16 eV) photons, electron–hole pairs are generated, separated by the application of an external bias, collected at the electrodes and added to the existing dark current generating a photocurrent (*I*_ph_ = *I*_light_ − *I*_dark_). This effect accounts for the great increase in conductivity of semiconductor photoelectric devices.^69^

The ON and OFF IR response characteristics of the device were investigated by fitting one cycle of 5 and 10 V photo–response curve for growth and decay rate constants (Fig. 8e and f respectively) using second order differential equations given by *I*(*t*)_growth_ = *I*_dark_ + *α* exp(*t*/*τ*_1_) + *β* exp(*t*/*τ*_2_) and *I*(*t*)_decay_ = *I*_dark_ + *χ* exp(−*t*/*τ*_1_) + *γ* exp(−*t*/*τ*_2_) respectively^45^ where *α*, *β*, *χ* and *γ* are scaling constants, *τ*_1_ and *τ*_2_ are time constants, *t* is the time for ON or OFF cycles and *I*_dark_ is the dark current. We then estimated the growth and decay time constants from these fits. At 5 V bias voltage, photocurrent rises rapidly within 2.12 s upon illumination followed by a slower component of 3.03 s before saturation. The average response time constant for this process given by *τ*_average_ = (*ατ*_1_ + *βτ*_2_)/(*α* + *β*)^45^ was found to be 2.16 s. Upon switching OFF the excitation laser the photocurrent followed a second order exponential relaxation process with an estimated time constant of 3.17 s followed by rapid decay of 1.97 s with an average time constant of 2.02 s before reaching the initial dark current. Similarly, when the bias voltage was 10 V, the growth and decay photoresponse followed a second order exponential relaxation process as in the case of 5 V. The growth time constants were 1.14 and 2.05 s with an average time constant of 1.17 s while the decay time constants were found to be 1.15 and 0.67 s with an average time constant of 1.08 s. Faster response/decay was obtained at 10 V bias voltage which is consistent with the power law and is attributed to better separation of electron–hole pairs.^45^ To compare the performance of our device with other photodetector devices, we determined the important figures of merit which characterize photodetectors such as sensitivity (*S*), responsivity (*R*_λ_), external quantum efficiency (EQE), and detectivity (D*). Sensitivity gives the switching ratio (SR) of the device and is determined by *S* = *I*_ph_/*I*_dark_ where *I*_ph_ = *I*_light_ − *I*_dark_ and *I* is the current.^45^ We found high sensitivity of *S* = 5.18% upon illumination under 1064 nm laser at power density of 200 mW cm^−2^. Responsivity indicates how the efficiency of the detector responds to the optical signal.^74^ It is defined as the photocurrent generated per unit power of incident light on the effective area^75^ and is given by *R*_λ_ = *I*_ph_/*P*_λ_*A* where *I*_ph_ is the photocurrent, *P*_λ_ is the power density and *A* is the effective area of the device.^45,76^ High responsivity indicates that a large photocurrent can be achieved under a relatively low optical input.^74^ Our device gave *R*_λ_ value of 0.75 and 1.54 mA W^−1^ at bias voltage of 5 and 10 V respectively with laser wavelength of 1064 nm and at power density of 200 mW cm^−2^. It is comparable to that shown by other photodetectors.^45^ External quantum efficiency defined as the measure of the number of photons absorbed to the number of incident photons was given by EQE = *hcR*_λ_/*qλ* where *h* is the Planck's constant, *c* is the speed of light, *R*_λ_ is responsivity, *q* is the electron charge and *λ* is the wavelength of the laser used.^45,68^ We found the EQE of device to be 0.09% and 0.18% at bias voltage of 5 and 10 V respectively under illumination of 1064 nm laser at 200 mW cm^−2^. The EQE, as expected from the power law, doubled as the bias voltage was doubled indicating that electron–hole pairs were efficiently separated at higher bias voltage. To determine the sensibility of the device to detect weak optical signals, detectivity was calculated from the relation; D* = *I*_ph_/[*P*_λ_(2*qI*_d_*A*)^1/2^] = *R*_λ_*A*^1/2^/(2*qI*_d_)^1/2^ where *R*_λ_ is responsivity, *A* is the effective area of the detector in cm^2^, *q* is the electron charge, *I*_d_ is the dark current, *P*_λ_ is the power density and *I*_ph_ is the photocurrent.^75–77^ Our device showed high detectivity of D* = 2.43 × 10^10^ and 3.53 × 10^10^ jones at bias voltage of 5 and 10 V respectively at 200 mW cm^−2^ power density under 1064 nm laser. We also calculated the photoconductive gain (*G*) defined as the ratio of the number of electrons collected per unit time (*N*_el_) to the number of absorbed photons per unit time (*N*_ph_) from the relation^78^*G* = *N*_el_/*N*_ph_ = *R*_λ_[1.24/*λ*(μm)*η*] = *τ*/*τ*_tr_ = *I*_ph_/*qF* where *R*_λ_ is responsivity of the detector, *λ* is the incident light wavelength, *η* is quantum efficiency, *τ* is hole (minority) lifetime, *τ*_tr_ is electron transit time, *I*_ph_ is photocurrent, *q* is the elementary charge, and *F* is the photon absorption rate and found it to be equal to *G* = 9.99 × 10^3^. All these results for *S*, *R*_λ_, EQE, D* and *G* exhibit the high performance of our VO_2_ films in optoelectronic devices and compare well with other photodetectors.^45,68,69,78^ The performance of this device is based on VO_2_ photoconductor which has a direct band gap. The performance of this device can further be exploited in future work and we believe that it can match or be better than IR commercial detectors fabricated from Si which is associated with an indirect band gap.

## 4. Conclusion

In summary, we have synthesized, characterized and fabricated an IR photodetector based on phase pure VO_2_(M1) thin film on quartz substrate by a simple cost-effective technique; ultrasonic nebulized spray pyrolysis of aqueous combustion mixture (UNSPACM). Phase purity was confirmed by XRD and Raman spectroscopic studies with an optical band gap of 0.75 eV. FTIR characterization revealed large thermal switchability of the film about 60% of the peak reflectivity across the transition temperature of 68 °C. The film showed a low reflectivity at low temperature and high reflectivity above the transition temperature. Electrical characterization showed a first order transition with a resistance change of four orders of magnitude with *T*_IMT_ of 68 °C. Hall-effect measurements revealed the n-type nature of VO_2_ thin films with room temperature Hall mobility, *μ*_e_ = 0.097 cm^2^ V^−1^ s^−1^, conductivity, *σ* = 0.102 Ω^−1^ cm^−1^ and carrier concentration, *n*_e_ = 5.36 × 10^17^ cm^−3^. Temperature dependent Hall-effect measurements showed an increase in *n*_e_ and *σ* by three orders of magnitude across the transition temperature. The fabricated IR photodetector exhibited a sensitivity of 5.18%, responsivity of 1.54 mA W^−1^, external quantum efficiency of 0.18%, detectivity of 3.53 × 10^10^ jones and photoconductive gain of 9.99 × 10^3^ upon illumination with a 1064 nm laser at a power density of 200 mW cm^−2^ and at bias voltage of 10 V. This strategy opens way for large scale synthesis of VO_2_ thin films for various applications in optoelectronic devices.

## Conflicts of interest

There are no conflicts to declare.

## Supplementary Material
